# Generating Rho-0 Cells Using Mesenchymal Stem Cell Lines

**DOI:** 10.1371/journal.pone.0164199

**Published:** 2016-10-20

**Authors:** Mercedes Fernández-Moreno, Tamara Hermida-Gómez, M. Esther Gallardo, Andrea Dalmao-Fernández, Ignacio Rego-Pérez, Rafael Garesse, Francisco J. Blanco

**Affiliations:** 1 Servicio de Reumatología, Instituto de Investigación Biomédica de A Coruña (INIBIC), Complexo Hospitalario Universitario de A Coruña (CHUAC), Sergas, Universidade da Coruña (UDC), A Coruña, Spain; 2 Departamento de Bioquímica, Instituto de Investigaciones Biomédicas “Alberto Sols” UAM-CSIC, Centro de Investigación Biomédica en Red de Enfermedades Raras (CIBERER), Madrid, Spain; Northwestern University, UNITED STATES

## Abstract

**Introduction:**

The generation of Rho-0 cells requires the use of an immortalization process, or tumor cell selection, followed by culture in the presence of ethidium bromide (EtBr), incurring the drawbacks its use entails. The purpose of this work was to generate Rho-0 cells using human mesenchymal stem cells (hMSCs) with reagents having the ability to remove mitochondrial DNA (mtDNA) more safely than by using EtBr.

**Methodology:**

Two immortalized hMSC lines (3a6 and KP) were used; 143B.TK-Rho-0 cells were used as reference control. For generation of Rho-0 hMSCs, cells were cultured in medium supplemented with each tested reagent. Total DNA was isolated and mtDNA content was measured by real-time polymerase chain reaction (PCR). Phenotypic characterization and gene expression assays were performed to determine whether 3a6 Rho-0 hMSCs maintain the same stem properties as untreated 3a6 hMSCs. To evaluate whether 3a6 Rho-0 hMSCs had a phenotype similar to that of 143B.TK-Rho-0 cells, in terms of reactive oxygen species (ROS) production, apoptotic levels and mitochondrial membrane potential (Δψm) were measured by flow cytometry and mitochondrial respiration was evaluated using a SeaHorse XFp Extracellular Flux Analyzer. The differentiation capacity of 3a6 and 3a6 Rho-0 hMSCs was evaluated using real-time PCR, comparing the relative expression of genes involved in osteogenesis, adipogenesis and chondrogenesis.

**Results:**

The results showed the capacity of the 3a6 cell line to deplete its mtDNA and to survive in culture with uridine. Of all tested drugs, Stavudine (dt4) was the most effective in producing 3a6-Rho cells. The data indicate that hMSC Rho-0 cells continue to express the characteristic MSC cell surface receptor pattern. Phenotypic characterization showed that 3a6 Rho-0 cells resembled 143B.TK-Rho-0 cells, indicating that hMSC Rho-0 cells are Rho-0 cells. While the adipogenic capability was higher in 3a6 Rho-0 cells than in 3a6 cells, the osteogenic and chondrogenic capacities were lower.

**Conclusion:**

Among the drugs and conditions tested, the use of d4t was the best option for producing Rho-0 cells from hMSCs. Rho-0 cells are useful for studying the role of mitochondria in hMSC differentiation.

## Introduction

Mitochondrial dysfunction is central to the pathogenesis of some monogenic syndromes. Examples of these syndromes include the MELAS syndrome (mitochondrial encephalomyopathy, lactic acidosis, and stroke-like episodes caused by mutation of mitochondrial transfer RNAs) [1,2] and Leigh’s disease (caused by mutations in genes related to oxidative phosphorylation) [1,3,4]. In addition, there is an emerging recognition that disordered mitochondrial dynamics contribute to the pathogenesis of complex diseases not classically considered to involve mitochondria; these diseases include cancer [5,6], cardiovascular disease [7,8,9], neurodegenerative diseases [10,11] and rheumatic diseases [12–15].

In the last century, immortal cell lines have been developed that are devoid of mitochondrial DNA (mtDNA) (Rho-0 (ρ0)) [16]. Rho-0 cells are highly valid tools to study human mitochondrial disorders because they can be used to develop a cytoplasmic hybrid (cybrid) model. This model is interesting because it allows the study of the real role of mtDNA single nucleotide polymorphisms (SNPs) with the same nuclear DNA background. Mitochondrial functions are controlled by both mtDNA and nuclear DNA; cybrids are useful for the difficult task of identifying whether the mitochondrial or nuclear genome is responsible for a particular mitochondrial defect. Cybrids are constructed by fusing a cell without a nucleus that harbors the mtDNA of interest with Rho0 cells in which endogenous mtDNA has been depleted. Cybrid cell lines have been successfully used to explore the contribution of mitochondrial dysfunction and mtDNA gene mutations to the pathogenesis of diseases, such as Parkinson's Syndrome. Because disease cybrids can be generated from patients at all stages of a disease, they provide a window into early stages of disease pathogenesis not available from pathological specimens. Therefore, Rho-0 cells represent an important tool for development of cellular models of disease, for studying the pathogenesis of some diseases, or to test the toxic effects of drugs.

The generation of Rho-0 cells is challenging and requires the use of an immortalization process followed by a lengthy culture in the presence of various low-dose drugs. Surprisingly, this does not ensure the complete depletion of mtDNA from the cells. The use of ethidium bromide (EtBr) is the most common and successful procedure to generate Rho-0 cells because EtBr possesses high capacity to intercalate into the mitochondrial double-stranded DNA, thereby interfering with enzymes of the replication machinery. However, mutagenic effects of EtBr on the nuclear genome cannot be excluded [17].

At present, most Rho-0 cells are obtained from tumor cells, with all the drawbacks that this entails [18–20]. Mesenchymal stem cells (MSCs) are stromal cells that were originally isolated from the adherent portion of bone marrow [21,22]. MSCs grow as spindle-shaped cells displaying a colony-forming capability in low density cultures and are non-hematopoietic and non-endothelial. MSCs can be propagated through multiple passages in cell culture and differentiate into the standard osteogenic, adipogenic and chondrogenic lineages in appropriate media [23].

Because of the adverse effects of EtBr and the inconvenience of using tumor cells to generate Rho cells, we conducted this experiment to attempt to improve these limitations. We first tested some reagents with the ability to remove mtDNA more safely than EtBr. To this end, we selected substances with the capacity to decrease the mtDNA copy number of cells by inhibiting mitochondrial oxidative phosphorylation, interfering with mtDNA replication, or inhibiting polymerase γ [24–27]. Subsequently, we replaced tumor cell lines with hMSCs from bone marrow. Although hMSCs are readily isolated but they have limited ability to expand under standard conditions or with increasing age [28,29] and rapidly senesce in culture with restricted multilineage differentiation capacity [30,31]. For this reason we used two immortalized hMSC lines, KP and 3a6 [32,33].

## Material and Methods

### Cell culture of hMSCs

Two immortalized human mesenchymal/stromal cell lines (hMSCs) were used in this work, the KP and 3a6 hMSC lines. Both hMSC lines were kindly provided by Dr Hung’s group.

KP cells were developed by Hung’s group using hMSC isolated from a bone marrow (BM) aspirate of a 61-year-old female donor in the Veteran´s General Hospital-Taipei. The KP cell line was immortalized using a retroviral vector transduction that expressed HPV16 E6/E7 [32]. KP cells were then transfected with phTERT-IRES2-EGFP to obtain the 3a6 cell line [33]. KP cells were cultured using the Mesenchymal Stem Cell Growth Medium Bullet Kit (LONZA, Biowhittaker, Viviers, Belgium), while 3a6 cell lines were grown in Dulbecco´s Modified Eagle´s Medium-Low Glucose (DMEM-LG) (GIBCO, Grand Island, NY, USA) supplemented with 10% fetal bovine serum (FBS), penicillin (100 U/ml), streptomycin (100 μg/ml) (P/S; Gibco, Paisley, United Kingdom) and 2 mM L-glutamine (GlutaMax, GIBCO).

The study was approved by the Ethics Committee of the Galician Health Administration.

### Rho-0 cell culture

The 143B.TK-Rho-0 cell line used in all the experiments as a reference control was kindly provided by Dr Garesse´s group. It was obtained from a human osteosarcoma cell line (143B.TK-) subsequently treated for 6–8 weeks with low dosage (50 ng/ml) EtBr. 143B.TK-Rho-0 cells were maintained in DMEM containing 10% FBS, P/S and 50 mg/ml uridine (Sigma-Aldrich, St. Louis, MO, USA). Rho-0 cells need specific supplementation with uridine to sustain their viability. The uridine satisfies the energy demand of these cells and possibly generates pyrimidine despite the inhibition of dihydroorotate dehydrogenase (DHODH), a key enzyme in the biosynthesis of pyrimidine.

In all cases the medium was changed twice a week and subculture was performed after the cells reached 80% confluency. Cells were maintained in a 5% CO2 and 90% humidified atmosphere at 37°C.

### Generation of hMSC Rho-0 cells

**Fig 1:**
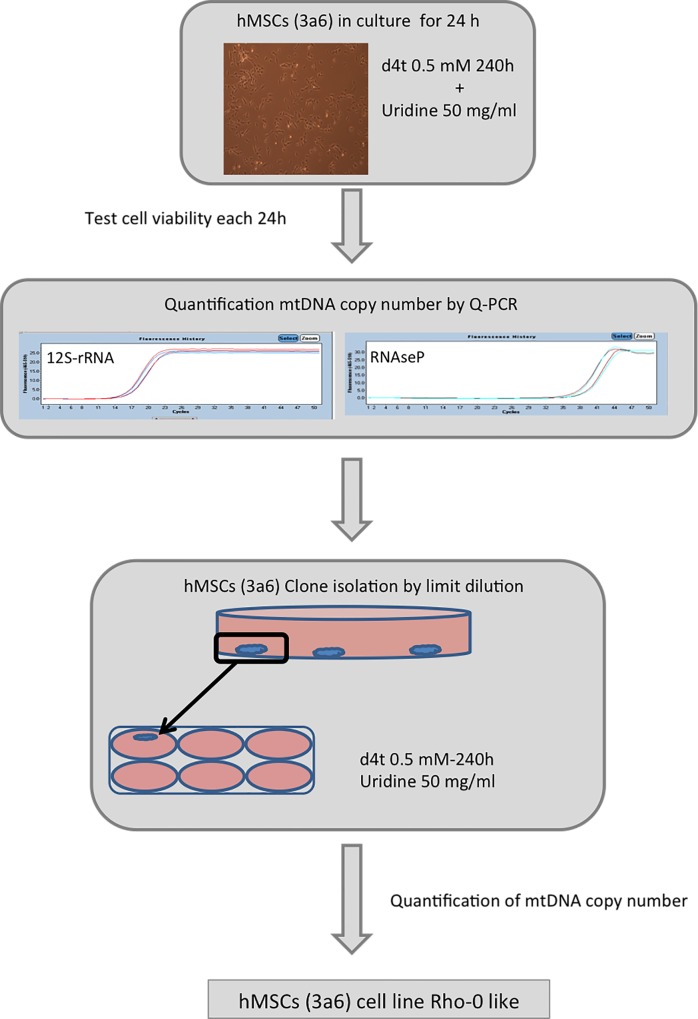
Work flow for generating Rho-0 Cells from human mesenchymal stem cells (hMSCs). d4t = Stavudine; mtDNA = mitochondrial DNA; Q-PCR = quantitative polymerase chain reaction; 12S-rRNA = mitochondrial12S ribosomal gene; RNAseP = nuclear gene.

Cells were plated in a 6-multiwell plate (MW-6; Corning Incorporated, Kennebunk, ME, USA) (8x104 cells per well) and cultured in the appropriate medium for each type, following the same procedure (**Fig 1**). Cells were maintained for 24 hours in the appropriate culture medium before the medium was supplemented with each tested condition: 3,8-Diamino-5-ethyl-6-phenylphenanthridinium bromide (Ethidium bromide, EtBr) (Sigma-Aldrich), Rhodamine 6g (Rd6g) (Invitrogen, Waltham, MA, USA), 1-methyl-4-phenylpyridinium (MPP^+^), Zidovudine (AZT, 3´-azido-3´-deoxythymidine) or Stavudine (d4t; 2'-3'-didehydro-2'-3'-dideoxythymidine) (Sigma-Aldrich) (**Table 1**). Culture media were always supplemented with uridine (50 mg/ml). Drug stocks were prepared following the manufacturers' instructions and replenished at each medium change. The stocks were stored at -20°C between doses and new stocks were prepared before each experiment.

**Table 1:** List of drugs analyzed to deplete mitochondrial DNA (mtDNA) in human mesenchymal stem cell lines (hMSCs).

Drug	Concentration	Time (Hours)
Etidium bromide (EtBr)	1 mM, 500 nM, 100 nM	168
Rhodamine-6g (Rd6g)	1 μg/ml, 3 μg/ml, 5 μg/ml	24–168
1-methyl-4-phenylpyridinium (MPP+)	25 μM, 0.5 mM, 1 mM	72
Zidovudine (AZT)	10 μM, 20 μM, 0.5 mM	96–240
Stavudine (d4t)	5 μM, 10 μM, 100 μM, 0.5 mM	96–240

In all the experiments, cells with no treatment were used as controls to determine the baseline levels of mtDNA for each cell type. Cells cultured with EtBr were used as positive controls to assess the capacity of the different cell lines to survive with very low levels of mtDNA. 143B.TK-Rho-0 cells were used as a reference for the mtDNA content characteristic of a standard Rho-0 line.

### DNA isolation and quantification of mtDNA copy number

Total DNA from untreated cells (5x10^5^) and cells cultured with the test compounds (1x10^5^) was isolated using a commercial kit (Qiagen, Manchester, UK), following the manufacturer's instructions.

The mtDNA content was measured by real-time polymerase chain reaction (PCR) using the LightCycler® 480 II (Roche Applied Science, Mannhein, Germany). The targeted genes were the mitochondrial12S ribosomal gene (12S-rRNA) and the nuclear gene RNAseP. Duplicate DNA samples were amplified in parallel in a final volume of 20 μL containing 50 ng DNA, 10 μL of LightCycler® 480 Sybr Green I Master (Roche Diagnostics),0.3 μM primers for 12S-rRNA (forward 5´-CCACGGGAAACAGCAGTGAT-3', reverse 5´-CTATTGACTTGGGTTAATCGTGTGA-3') and 0.3 μM RNAseP primers (forward: 5´-GCACTGAGCACGTTGAGAGA-3'; reverse: 5´-CCAGTCGAAGAGCTCCAGA-3') with H2O to reach the final volume. The mixture was amplified by 50 cycles at 95°C for 10 seconds, 60°C for 15 seconds and 72°C for 10 seconds; with a final extension of 72°C for 1 minute. The specificity of all reactions was determined by a melting point curve analysis using one cycle at 95°C for 5 seconds and 65°C for 1 minute followed by a heating step at 97°C with continuous fluorescence acquisition. For determining mtDNA copy number, an independent standard curve was generated for each gene (12S-rRNA and RNAseP). The total mtDNA copy number was determined from the Ct values and was extrapolated into the external standard curve. The concentration for each gene was obtained in the analyzed samples. MtDNA copy number values were expressed by the ratio 12S rRNA/RNAseP. To normalize the values between all experiments, we established the mtDNA copy number using the control cells (cells without treatment) as 100%.

### Clone selection

For the 3a6 cell line, clone selection was performed in the presence of d4t at 0.5 mM over a 240-hour period (**Fig 1**). Cells were plated in a P-100 dish at very low density; 90 cells were plated (limit dilution) in DMEM-LG supplemented with 10% FBS, P/S, 0.5 mM d4t and 50 mg/ml uridine. After 24 hours cells were examined under an optical microscope and isolated cells were marked to be followed for 2–3 weeks in culture. When a single colony was formed, it was transferred to a MW-6 well. Clones were treated for 192–240 hours with the selective medium (DMEM-LG supplemented with 10% FBS, P/S, 50 mg/ml uridine and d4t 0.5 mM). At the end of this second selection time, mtDNA levels were determined and compared with those obtained for 143B.TK- Rho-0 cells (**Fig 1**).

These treated cells and their Rho-0 clones were stored in liquid nitrogen for further use.

### Phenotypic characterization of cells by flow cytometry

Cells were harvested, centrifuged, washed, and counted prior to flow cytometry. Cells were incubated with 12 antibodies (CD29, CD34, CD45, CD69, CD73, CD90, CD105, CD106, CD166, CD271, STRO and SSEA4) at optimal amounts added to each tube for 1 h at 4°C. A control tube for each of the chromogens received equivalent amounts of isotype standards. Rabbit polyclonal anti-mouse IG-FITC (Dako, Barcelona, Spain) was used as a secondary antibody when necessary. 2x10^4^ cells per assay were analyzed on a flow cytometer (FACsCalibur, Becton Dickinson, Madrid, Spain). Results were expressed as mean percentage of positive cells in three individual experiments (mean ± SD).

### Detection of cellular reactive oxygen species (ROS) production

Cells were plated at a density of 8x10^4^ cells per well in MW-6 plates for 24 hours, them the medium was depleted and cells were incubated for one hour prior to the incubation with 2,7-dichlorodihydrofluorescein diacetate at 10 μM (DCFH-DA, Sigma-Aldrich) for 30 min at 37°C in darkness. Cells were harvested by trypsin release and resuspended in saline solution prior to analysis by flow cytometry. 10^4^cells per assay were measured by flow cytometry. Data were analyzed using CellQuest software (Becton Dickinson). Results were expressed as median of fluorescence (AU) and represented three independent experiments.

### Detection of mitochondrial membrane potential (MMP, Δψm) and apoptotic cells

To determine mitochondrial membrane potential, cells were plated at 8x10^4^ cells per well in MW-6 plates for 48 hours in DMEM with 10% FBS. Cells were harvested, washed and resuspended phosphate-buffered saline (PBS). To establish the mitochondrial membrane potential, 5 μl of 1,1´,3,3,3´-hexamethylindodicarbo-cyanine iodide (DilC1(5) ImmunoStep, Salamanca, Spain) was added and incubated for 15 min at 37°C, 5% CO2. To measure the MMP, we used a lipophilic fluorescent stain for labeling membranes, DilC1(5). This dye accumulates primarily in those mitochondria having active mitochondrial membrane potentials. Staining decreases as the mitochondrial membrane potential is reduced.

For apoptosis, the cells were incubated in the presence of staurosporine at 2 μM for 2 hours. The basal and staurosporine-conditioned cells were washed and resuspended in 1 X annexin-binding buffer followed by 5 μl of Annexin V-FITC and 5 μl of propidium iodide (PI) (ImmunoStep) added to each 100 μl of cell suspension. The cells were then incubated at room temperature for 15 minutes in darkness. Following incubation, 400 μl of 1X annexin-binding buffer was added prior to analysis by flow cytometry within one hour. 10^4^ cells per assay were measured on a flow cytometer and data obtained were analyzed by CellQuest software (Becton Dickinson). Analysis of apoptosis was performed by counting cells stained simultaneously with Annexin V–FITC and PI. This allowed the discrimination of intact cells (Annexin V-FITC and PI negative) from cells in the early apoptotic state (Annexin V-FITC positive and PI negative) and late apoptotic state (Annexin V-FITC and PI positive).

Results are expressed as percent of positive cells for each dye and represent the mean of three independent experiments (mean ± SD).

### Mitochondrial respiration

Oxygen consumption (OCR, pmoles/min), an indicator of mitochondrial respiration, was determined by direct measurement with a SeaHorse XFp Extracellular Flux Analyzer instrument (Seahorse Bioscience, Agilent Technologies, Santa Clara, CA, USA). 2x10^4^ cells per well were seeded in XF cell culture microplates (Seahorse Bioscience, Agilent Technologies) and incubated at 37°C with 5% CO_2_. The next day, the cells were washed with XF assay medium supplemented with 10 mM glucose and 1 mM pyruvate and placed in a 37°C incubator without CO_2_ for 1 hour. OCR was determined following the manufacturer’s instructions. Briefly, for the mitochondrial stress test, ports were loaded with 2 μM oligomycin, an ATP synthase inhibitor, 1 μM carbonyl cyanide p-trifluoromethoxyphenyl-hydrazone or FCCP, an oxidative phosphorylation uncoupler, and, finally, a mixture of 2 μM Rotenone, a respiratory complex I inhibitor and 4 μM Antimycin A, a respiratory complex III inhibitor, was added. Real time OCR was averaged and recorded three times during each conditional cycle.

### Osteogenic, adipogenic, and chondrogenic differentiation

For osteogenic and adipogenic differentiation, 3x10^3^ cells were seeded in wells of a MW-6 plate and cultured in growth medium for 24 hours. Osteogenesis was induced by culturing the cells in Osteogenic Differentiation Medium (Lonza, Biowhittaker). Adipogenesis was induced by culturing cells in hMSC Commercial Adipogenic Differentiation Medium (Lonza, Biowhittaker), following the manufacturer's instructions. Cultures were alternated between induction and maintenance medium every 3 days. Both differentiation processes were maintained for 9 days.

Chondrogenesis was assessed using the micropellet formation (2.5x10^4^) technique [34], with some modifications. 3a6 and 3a6 Rho-0 cells were detached using trypsin and centrifuged at 300xg for 10 minutes. The resulting pellet was cultured for 21 days in hMSC Commercial Chondrogenic Differentiation Medium (Lonza, Biowhittaker) supplemented with 10 ng/ml transforming growth factor-β3 (TGFβ-3) (Prospec, Ness-Ziona, Israel). During this process the culture medium was changed every 3 days.

### Staining neutral lipids

To confirm adipogenic differentiation capacity, 3a6 cells without treatment (3a6-wt) and 3a6 Rho-0 cells were cultured in adipogenic differentiation medium and the cells were then fixed in 4% paraformaldehyde (Sigma-Aldrich) for 10 minutes at 4°C, followed by double-staining for 30 minutes with a 1:10000 dilution of a neutral lipid dye, 4,4-Difluoro-2.3,5.6-bis-tetramethylene-4-bora-3a,4a-diaza-s-indacene (LD540), kindly provided by Dr Thiele [35]. This step was followed by 5 minutes incubation with the nuclear dye 2′-(4-Ethoxyphenyl)-5-(4-methyl-1-piperazinyl)-2,5′-bi-1H-benzimidazole trihydrochloride (Hoechst 33258. Sigma-Aldrich). After washing with PBS, cover slips were mounted on microscopy chamber slides using Glycergel (Dako). Fluorescence was visualized and photographed under fluorescence microscopy at 20X (Olympus BX61).

### RNA extraction and first strand cDNA synthesis

Total RNA from cell cultures and micropellets was extracted using Trizol® reagent (Invitrogen), following the manufacturer's protocol. For each sample, 1μg of total RNA was reversed transcribed using the SuperScript® ViloTM Master Mix (Invitrogen), following the manufacturer's instructions.

### Quantitative real-time-PCR (qRT-PCR)

Real-time PCR utilized a LightCycler 480-II Instrument (Roche, Mannheim, Germany) with TaqMan Universal Master Mix (Applied Biosystems). Analysis of the results was carried out using Qbase+ version 2.5 software (Biogazelle, Gent, Belgium). Gene expression in some cases was calculated relative to the housekeeping gene (RPL13A) and in other cases relative to the basal condition. Sequence primers, probe and PCR conditions can be provided upon request.

### Statistical analysis

Appropriate statistical analyses were performed using GraphPad Prism v6 software. The results are reported as mean ± SD. A p value less than 0.05 was considered significant.

## Results

### Generating Rho-0 cells from 3a6 human immortalized MSCs

**Fig 2:**
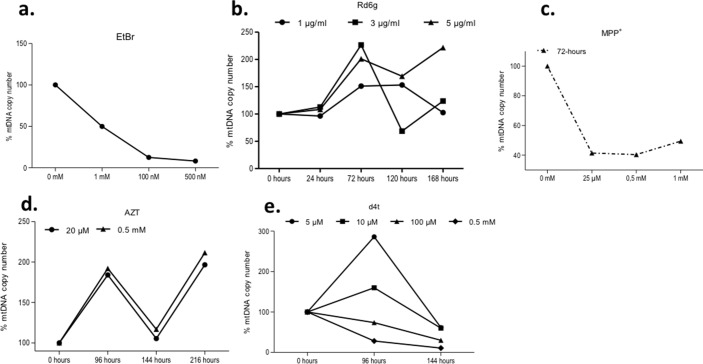
3a6 cell line response to different substances capable of depleting the levels of mitochondrial DNA. All mtDNA copy numbers are expressed as percentages comparing each value with untreated cells valued at 100% (**a**). Treatment with ethidium bromide (EtBr) at three different concentrations (1 mM, 100 nM and 500 nM) for 240 hours. The maximum effectiveness of this treatment was at 100 nM. (**b**). Treatment with Rhodamine 6g at 1, 3 and 5 μg/ml for 24, 72, 120 and 168 hours. (**c**). Treatment with 1-methyl-4-phenylpyridinium (MPP^+^) at 25 μM, 0.5 mM and 1 mM for 72 hours. (**d**). Treatment with Zidovudine (AZT) at 20 μM and 0.5 mM, for 96, 144 and 216 hours. AZT increased mtDNA content. (**e**). Treatment with Stavudine (d4t) at 5, 10 and 100 μM and 0.5 mM for 96 and 144 hours. The analysis of mtDNA copy numbers reflect that treatment with the highest concentration (0.5 mM) for 144 hours decreased the levels of mtDNA in treated cells nearly 98% compared to those of untreated cells. 8x10^4^ cells were plated in each experiment for treatment with a reagent. The figures represent at least three independent experiments.

Because this is the first time that a hMSC cell line was used to generate cells with depleted mtDNA, we first tested survivability, as well as capability to become Rho-0 cells, using classic methodology with EtBr. The results showed that the 3a6 cell line cultured with EtBr at 100 nM for a short time, 240 hours, had a 8.17% decrease of mtDNA copy number compared to 3a6 cells without treatment (3a6-wt, mtDNA copy level was established at 100%) (**Fig 2A**). These results demonstrate the capacity of the 3a6 cell line to deplete its mtDNA and survive in culture in the presence of uridine.

The next set of experiments studied the ability of other reagents less toxic than EtBr to reduce mtDNA copy number. 3a6 cells cultured in the presence of three different Rd6g concentrations (1, 3 and 5 μg/ml) for 168 hours showed a small decrease in mtDNA content (**Fig 2B**). Rd6g at 3 μg/ml for 120 hours yielded the greatest decrease in the percentage of mtDNA content, 68.5%. The highest concentration, 5 μg/ml, proved to be the least effective, with the levels of mtDNA actually increasing compared to cells without treatment (**Fig 2B**).

The lowest concentration of MPP^+^, 25 μM, at 72 hours of culture reduced the percentage of mtDNA copies to 41.41% compared to the control. The other two concentrations, 0.5 mM and 1 mM, produced similar results at the same time (**Fig 2C**). A culture time longer than 72 hours with MPP^+^ induced cell death by necrosis at all concentrations (data not shown).

Interestingly, the capacity of the two nucleosides reverse transcriptase inhibitors (NRTIs) selected differed. Both AZT concentrations, 20 μM and 0.5 mM, at all culture times, increased the mtDNA levels and both concentrations showed similar effects on mtDNA copy number (**Fig 2D**). However, incubation of cells with Stavudine (d4t) yielded different results. Low concentrations (5 and 10 μM) did not produce a decrease in the mtDNA copy number, but high concentrations, 100 μM and 0.5 mM, incubated for 144 hours decreased mtDNA copy number to 10.84% compared to that of the control (**Fig 2E**). The most effective concentration and culture times for reducing mtDNA copy number in 3a6 hMSCs are summarized in **Table 2**.

**Table 2:** Summary of the most effective reagent reducing mitochondrial DNA (mtDNA) copy number in 3a6 human mesenchymal stem cells (hMSCs).

Drug	Concentration	Time (Hours)	3a6% mtDNA copy number
Etidium bromide (EtBr)	100 mM	168	8.15
Rhodamine-6g (Rd6g)	3 μg/ml	120	84.97
1-methyl-4-phenylpyridinium (MPP+)	25 μM	72	41.41
Zidovudine (AZT)	0.5 mM	216	221.55
Stavudine (d4t)	0.5 mM	144	2.86

**Fig 3:**
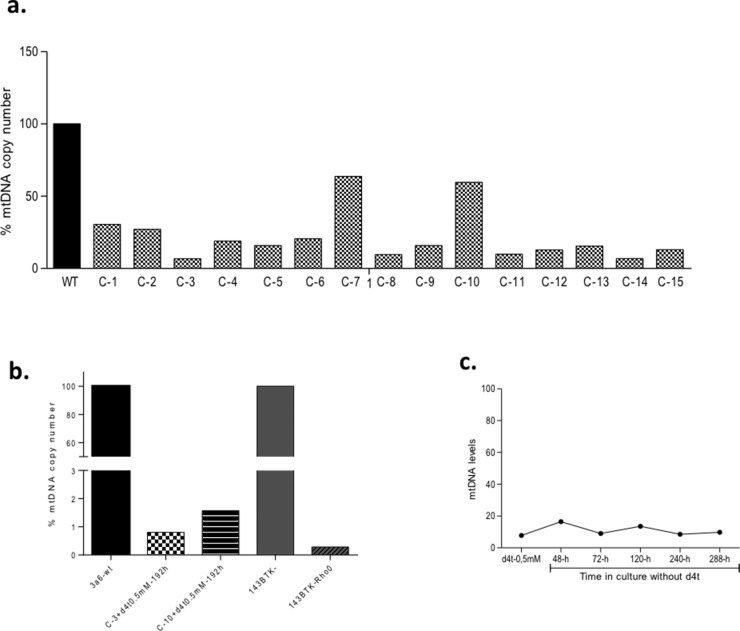
Clone Selection. (**a**). The mitochondrial DNA (mtDNA) copy number of 15 clones isolated by limit dilution from 3a6 cells treated with Stavudine (d4t) at 0.5 mM for 240 hours. Data for clones are expressed as percentages of untreated cells [without treatment (wt), value 100%]. (**b**). mtDNA copy number of two selected clones, one with low mtDNA (C-3; 1.57%) and the other with a high value for this parameter (C-10; 0.80%). Both clones were re-treated with d4t at 0.5 mM for 192 hours with mtDNA copy number expressed as a % of that of the 3a6 parental line. In this graphic, the %mtDNA copy number for 143B.TK- Rho-0 is also represented in relation to that of its parental cell line (143B.TK-). (**c**). This graph shows the % of mtDNA copy number for C-3 cultured in DMEM without d4t for 288 hours. The results show the stability of the mtDNA copy number during culture without the drug.

The values obtained to this point come from a pool of cells; therefore we performed clone isolation. Cells from the 3a6 cell line were cultured in the presence of d4t at 0.5 mM for about 96 hours before selection of each clone. First, we isolated 15 clones and analyzed the mtDNA copy number from each. The results showed variability in the mtDNA copy number ranging from 67.79% (clone-7) to 10.76% (clone-3), compared to 3a6 cells without treatment (3a6-wt) (**Fig 3A**). To decrease the mtDNA copy numbers detected in 3a6 clones to values similar to those described for other Rho-0 cells, such as 143B.TK- Rho-0, two 3a6 clones were selected. One, C-10, had a high mtDNA copy number; 63.68%, and the other, C-3, the lowest value for mtDNA copies, 10.76%. Both clones were cultured with uridine (50 mg/ml) and d4t (0.5 mM) for an additional 192 hours prior to analysis of the mtDNA copy number. The results showed that both selected clones had obtained a low number of copies when compared to their parental line, 0.80% for C-3 and 1.57% for C-10, when 3a6 was assigned the mtDNA copy number value of 100% (**Fig 3B**). These values are characteristic of Rho-0 cell lines, as shown in the same Fig where the % of mtDNA copy number in the 143B.TK-Rho-0 line (0.284%) is compared to that of its parental cell (143B.TK-) (100%).

To test the ability of the selected clones to grow in culture medium without d4t and maintain the mtDNA levels of a Rho-0 cell line, we cultured C-3 cells (pre-treated for 192 h with d4t at 0.5 mM) for 288 hours in a culture medium supplemented only with uridine. We then isolated the total DNA at 48, 72, 120, 240 and 288 hours of culture and assessed the mtDNA copy number. The results showed that these cells maintained low levels of mtDNA (**Fig 3C**). These results indicate that the clones isolated from 3a6 are Rho-0 cells and could be used to construct transmitochondrial cybrids. The C-3 clone was selected for use in further experiments.

**Fig 4:**
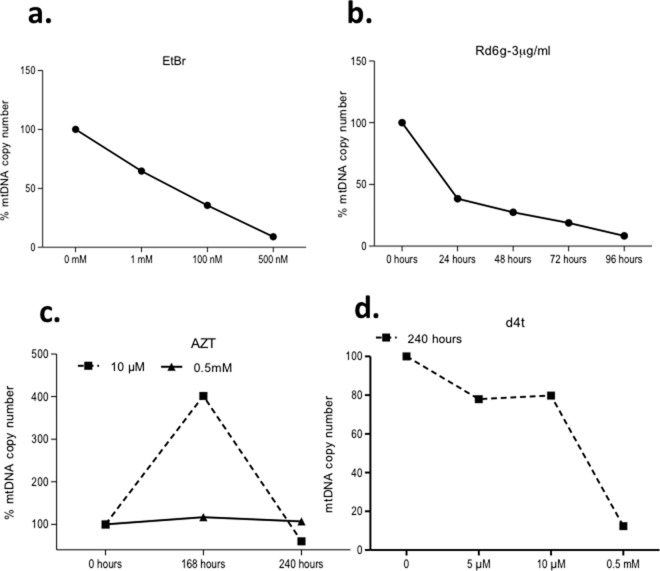
The response of the KP cell line to different substances that deplete mitochondrial DNA. All mtDNA copy numbers are expressed as percentages compared to those of untreated cells valued at 100%. (**a**). Treatment with ethidium bromide (EtBr) at three different concentrations, 1 mM, 500 nM and 100 nM, for 240 hours. (**b**). Treatment with Rhodamine6g (Rd6g) at 3 μg/ml for 24, 48, 72 and 96 hours; the positive response started at 48 hours. (**c**). Treatment with Zidovudine (AZT) at 10 μM and 0.5 mM for 168 and 240 hours. (**d**). Treatment with Stavudine (d4t) at three concentrations, 5 μM, 10 μM and 0.5 mM, for 240 hours. 8x10^4^ cells were plated in each experiment for treatment with different agents. The figures represent at least two independent experiments.

Because all reagents do not possess the same capability for removing mtDNA in different cell lines, we examined the ability of these reagents in another hMSC cell line generated from the same cell line donor as 3a6. We used the KP cell line to evaluate the efficiency of the compounds tested on 3a6. We first determined the ability of KP cells to become Rho-0 cells. The results indicated that KP cells incubated with EtBr at 500 nM for 168 hours showed decreased levels of mtDNA copy number, 8.93%, compared to cells without treatment (KP control at 100%) (**Fig 4A**). Rd-6g (3 μg/ml) lowered the mtDNA content beginning on the second day of exposure, the level of mtDNA at 96 hours being 8.34% (**Fig 4B**). The capacity of NRTIs in KP cells was similar to that in 3a6 cells. Culture with AZT produced mtDNA levels above 100%, except for 10 μM at 240 hours, at which concentration and time mtDNA levels decreased to near 60% (**Fig 4C**). Three concentrations of d4t were tested (5 μM, 10 μM and 0.5 mM) over 240 hours of culture and mtDNA levels decreased to 12.38% at 0.5 mM concentration (**Fig 4D**). The most effective concentration and time of culture of the conditions tested to reduce mtDNA copy number in the KP mesenchymal stem cell line are summarized in **Table 3**.

**Table 3:** Summary of the most effective reagent, in KP human mesenchymal stem cells (hMSCs).

Drug	Concentration	Time (Hours)	KP % mtDNA copy number
Etidium bromide (EtBr)	500 mM	168	8.93
Rhodamine-6g (Rd6g)	3 μg/ml	96	8.34
Zidovudine (AZT)	10 μM	240	59.68
Stavudine (d4t)	0.5 mM	240	12.38

### Characterization of Rho-0 hMSC cells

**Fig 5:**
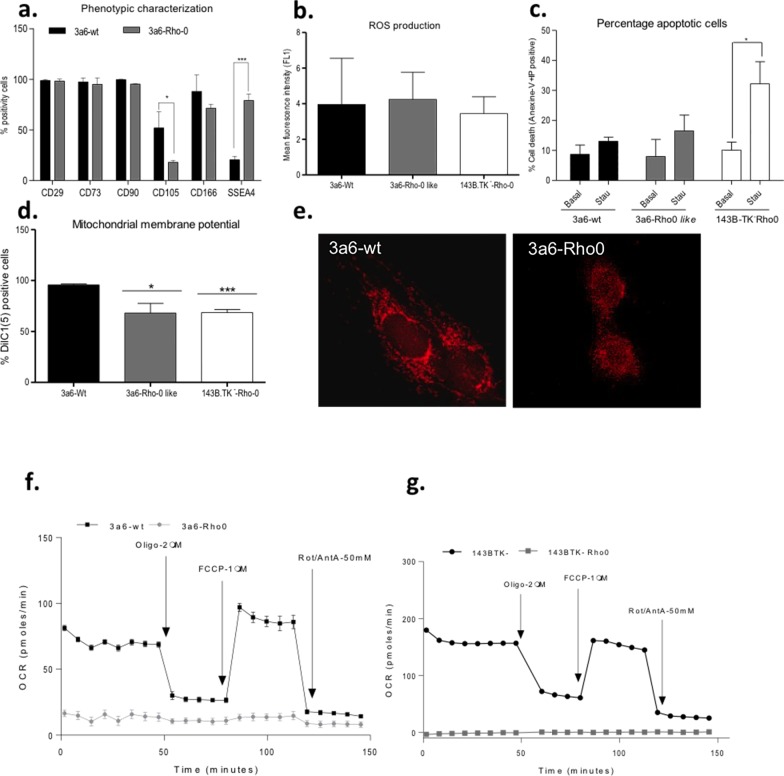
Characterization of 3a6 cells without treatment (3a6-wt) and 3a6 Rho-0 like cells. (**a**). Phenotypic characterization is represented as the percentage of positivity for CD29, CD73, CD90, CD105, CD166 and SSEA4 for both cell types. (**b**). Cellular levels of reactive oxygen species (ROS) in both cell types and in 143B.TK-Rho-0 cells (used as a reference for a typical Rho-0 line). Total ROS production was measured with 2,7-dichlorodihydrofluorescein diacetate (DCFH-DA); data are expressed as mean fluorescence intensity. (**c**). Apoptosis measured with Annexin-V-FIT: data are expressed as percentage of positive cells for Annexin-V and propidium iodide (PI) in basal conditions and culture in presence of Staurosporine (Stau) at 2 μM for 2 hours. (**d**). Mitochondrial membrane potential (Δψm) measured with DilC1(5) [1,1´,3,3,3´-hexamethylindodicarbo-cyanine iodide], data are expressed as percentages of cells that were positive for DilC1(5) fluorescence. (**e**). Mitochondrial network in 3a6-wt and 3a6-Rho 0 cells incubated with 250 nM MitoTraker Red solution for 30 min in a 37°C incubator. The cells were fixed with 4% paraformaldehyde and counterstained with Hoechst-33258 nuclear dye. The cells were photographed with a confocal microscope Nikon AR-1. (**f-g**) The mitochondrial respiration [oxygen consumption (OCR)] pattern was obtained using a SeaHorse XFp for 3a6-wt and 3a6 Rho-0 cells (**f**) and for 143B.TK- and 143B.TK-Rho-0 cells (**g**). All data were obtained from three independent experiments, expressed as mean ±SD and analyzed by the unpaired t-test (*, p≤0.05; *** p<0.001).

To assess whether 3a6 Rho-0-like cells maintain the stem properties of 3a6-wt cells, a phenotypic characterization was developed. FACS analysis demonstrated that 3a6-wt and 3a6 Rho-0 cells showed a similar cell-surface receptor pattern (**Fig 5A**). Both types of cells were negative for CD34, CD45, CD69, CD106, CD271 and STRO antigens (data not shown). In both 3a6-wt and 3a6 Rho-0-like cells nearly 95% of the cells were positive for CD29, CD73, and CD90. Significant differences existed for CD105 (3a6-wt: 51.96±16.07%; 3a6 Rho-0-like: 17.99±1.80%, p<0.05) and SSEA4 (3a6-wt: 20.45±3.52%; 3a6 Rho-0-like: 79.01±3.92%, p<0.0001).

The assessment of ROS production showed no significant differences between 3a6-wt, 3a6 Rho-0 and 143B.TK- Rho-0 cells (**Fig 5B**). While levels of apoptosis did not differ in numbers of positive cells between basal conditions and Staurosporine exposure in 3a6-wt and 3a6 Rho-0 cells, for 143B.TK- Rho-0 cells the difference was statistically significant (basal: 10.11%±2.67; Staurosporine 32.20%±7.36, p<0.05) (**Fig 5C**).

The mitochondrial membrane potential (Δψm) of 3a6 Rho-0 cells showed a significant decrease compared to that of 3a6-wt cells (3a6-wt: 95.66±1.86%; 3a6 Rho-0: 68.11±1,62%; p≤0.05). Similarly, the Δψm of the 143.B TK- Rho-0 cell line significantly decreased compared to that of 3a6-wt cells (3a6-wt: 95.66±1.86%; 143B. TK- Rho-0: 68.50±5.37%; p≤0.001) (**Fig 5D**).

The mitochondrial network was broken in the 3a6 Rho-0 cells and mitochondria were rounded and surrounded the nucleus when compared with 3a6-wt cells (**Fig 5E**). The 3a6 Rho-0 cells had a lower basal OCR and did not respond to the administration of oligomycin, FCCP and rotenone/antimycin while parental cells showed standard values. These results are similar to those found for 143B.TK- and 143B.TK-Rho-0 cells (**Fig 5F and 5G**).

### Differentiation capacity of Rho-0 hMSCs

**Fig 6:**
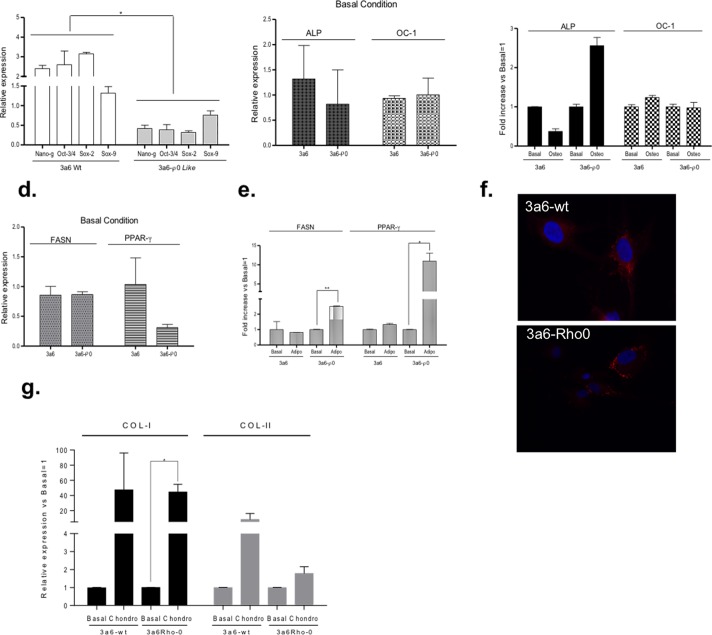
Osteogenic, adipogenic and chondrogenic differentiation in 3a6 without treatment (3a6-wt) and 3a6 Rho-0 cells. (**a**) Gene expressions implicated in the non-differentiation stage: Nano-HomeoBox (Nano-g), POU Class 5 Homeobox 1(Oct 3/4) and SRY (Sex determining region)-Y-box 2 (Sox-2) and SRY (Sex determining region)-Y-box 9 (Sox-9) in 3a6-wt and 3a6 Rho-0 cells. (**b**). Gene expressions implicated in the osteogenic process: alkaline phosphatase-4 (ALP) and osteocalcin-1 (OC-1) in basal condition in 3a6-wt and 3a6 Rho-0 cells. (**c**). Fold increase in the gene levels of ALP and OC-1 during osteogenic induction for 9 days; data are expressed relative to the basal condition (basal condition equal to 1). (**d**). Basal gene expressions implicated in the adipogenic process: fatty acid synthase (FASN) and peroxisome proliferator-activated receptor gamma (PPAR-γ) in 3a6-wt and 3a6 Rho-0 cells. (**e**). Fold increase in the gene levels of FASN and PPAR-γ following 9 days under adipogenic differentiation; data are expressed relative to the basal condition (basal condition equal to 1). (**f**). LD540, which stains lipid droplets, was used to measure adipogenic differentiation in 3a6-wt and 3a6 Rho-0 cells. Cells were cultured in standard medium (control) and in adipogenic medium for 9 days. Cells were fixed and double-stained with LD540 (red) for lipid droplets and Hoechst 33258 (blue) for DNA. Fluorescence was visualized with Olympus BX61 fluorescence microscopy and photographed at 20X. (**g**). Fold increase in the gene levels of CoL-I and CoL-II in the micropellet model following 21 days under chondrogenic differentiation; data are expressed relative to the basal condition (basal condition equal to 1). All data were obtained from three independent experiments, expressed as mean ±SD and analyzed by the unpaired t-test (*, p≤0.05; ** p<0.005). Basal, without induction. Osteo (Osteogenic), Adipo (Adipogenic), Chondro (Chondrogenic) cells grown in the presence of indicated medium.

To evaluate the multipotential capacity of Rho-0 hMSCs, we examined the expression of Nano-HomeoBox (Nano-g), POU Class 5 Homeobox 1 (Oct 3/4), SRY (Sex determining region)-Y-box 2 (Sox-2) and SRY-box 9 (Sox-9). The results of the q-PCR analyses revealed that the expression levels of the four genes in 3a6-Rho-0 cells were significantly lower than those in 3a6-wt cells (p≤0.05) (**Fig 6A**).

Another set of experiments was designed to study how reducing the expression of the above mentioned four genes affects the capacity of 3a6 Rho-0 cells to differentiate into osteocytic, adipocytic and chondrocytic cellular lineages.

Basal expressions of two genes involved in osteogenesis, alkaline phosphatase-4 (ALP) and osteocalcin-1 (OC-1), did not show any significant difference between 3a6 Rho-0 and 3a6-wt cells (**Fig 6B**). However, their expression after 9 days in culture with specific differentiation medium showed that ALP expression was higher in 3a6 Rho-0 than 3a6-wt cells (0.575±0.035-fold for 3a6-wt and 1.701±0.156-fold for 3a6 Rho-0). Expression levels of OC-1 after osteogenesis induction did not show significant differences between the two cell types (**Fig 6C**).

Basal expression of the fatty acid synthase (FASN) gene was similar in both 3a6-wt and 3a6-Rho-0 cells. The basal expression of peroxisome proliferator-activated receptor gamma (PPAR-γ) was higher in 3a6-wt than 3a6 Rho-0 cells, but not significantly so (**Fig 6D**). Interestingly, in 3a6-wt cells, the induction of adipogenesis differentiation did not modify the expressions of FASN and PPAR-γ (0.558±0.13-fold for FASN and 0.522±0.113-fold for PPAR-γ). However, differentiated 3a6 Rho-0 cells showed a significant increase in the levels of both genes (FASN: 2.45±0.14-fold, p≤0.005; PPAR-γ: 9.334±1.32-fold, p≤0.01) (**Fig 6E**). These results were confirmed by staining cells with LD540; cells cultured in adipogenic medium showed more neutral lipid in 3a6 Rho-0 cells than in 3a6-wt cells (**Fig 6F**).

To analyze the capacity of these cells to differentiate into chondrocytes, micropellet formation under chondrogenic differentiation was employed and the gene expression levels of CoL-I and CoL-II were evaluated. The results showed that chondrogenic differentiation increased the levels of CoL-1, but with statistical significance only in the case of 3a6 Rho-0 cells (44.38±10.28, p≤0.05). The levels of CoL-II increased more in 3a6-wt than in 3a6 Rho-0 cells (**Fig 6G**).

**Fig 7:**
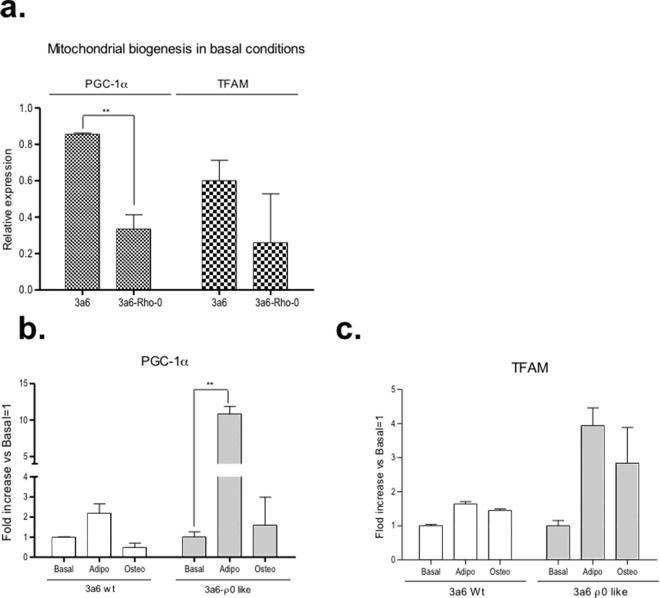
mRNA expression of mitochondrial biogenesis genes in 3a6 without treatment (3a6-wt) and 3a6 Rho-0 cells. (**a**). To analyze mitochondrial biogenesis, peroxisome proliferator-activated receptor gamma coactivator 1-alpha (PGC-1α) and transcription factor A, mitochondrial (TFAM) gene expression levels were determined under basal conditions. (**b**). Fold increase of PGC-1α gene levels during osteogenic and adipogenic induction. and (**c**). TFAM gene levels during osteogenic and adipogenic induction. Data are expressed relative to basal condition (basal condition equal to 1). All data were obtained from two independent experiments, expressed as mean ± SD and analyzed by the unpaired t-test (* p<0.05; ** p<0.005). Basal, in standard medium, Adipo (Adipogenic) and Osteo (Osteogenic); all experiments were developed during 9 days in each specific differentiation culture medium.

To examine the extent of mitochondrial activity involvement in the cellular differentiation process, the expression levels of two genes indicative of mitochondrial biogenesis, peroxisome proliferator-activated receptor gamma co-activator 1-alpha (PGC-1α) and transcription factor A mitochondrial (TFAM) were determined. The basal expression level of both genes was similar in 3a6-wt and 3a6 Rho-0 cells, but the basal genetic expression of PGC-1α was significantly higher in 3a6-wt than in 3a6 Rho-0 cells (p<0.01) (**Fig 7A**).

The gene expression level of PGC-1α after 9 days in culture with specific adipogenic medium in comparison with cells cultured in basal medium showed that its expression was significantly higher in 3a6 Rho-0 adipogenic than in basal condition (3a6 Rho-0-Adipo: 10.82±1.082-fold vs. 3a6 Rho-0-Basal p≤0.005) (**Fig 7B**). Similar results (but not significant) were obtained for TFAM gene expression (**Fig 7C**).

## Discussion

The first successful isolation of human Rho-0 cells used long-term exposure to EtBr of cells from the 143B.TK- cell line [16]. These authors found that growth of cells obtained by this method is uridine- and pyruvate-dependent because of the absence of a functional respiratory chain. To date, most Rho-0 cells used to construct cybrids have been generated following this procedure. We have, in this work, used EtBr to evaluate the capacity of cells of a hMSC line to become Rho-0 cells and survive. Our results confirm the capability of EtBr to deplete mtDNA in hMSCs and, furthermore, the ability of these depleted cells to survive with very low copies of mtDNA in culture containing uridine. However, EtBr is an intercalating agent that causes negative effects in the nuclear genome; therefore, we consequently analyzed other compounds with the aim of reducing the number of mtDNA copies with less toxicity. We examined the karyotype of these depleted cells and have shown that the compounds used to produce 3a6 Rho-0 cells do not induce change in the number and in the size of chromosomes. Both 3a6-wt and 3a6 Rho-0 cell types have similar chromosome conformation and their karyotypes are similar (data are not presented).

The first compound tested was Rhodamine 6g (Rd6g), which, in vivo penetrates into the cell and binds to the inner membranes of the mitochondria, decreasing mitochondrial enzymatic activity. This inhibits oxidative phosphorylation and reduces the number of intact and metabolically active mitochondria within the cell [24]. Rd6g has been used to remove mitochondrial DNA from cells in culture for the construction of cybrids or hybrids using hamster [36], mouse [37] and human skin fibroblasts [38]. Our results showed that Rd6g is less effective for removing mtDNA in hMSC cell lines, although in KP cells Rd6g produced a larger decrease of mtDNA copy number, but this reduction was not sufficient to generate Rho-0 cells. It is possible that Rd6g causes rapid and irreversible damage to mitochondria without removing mtDNA [38,39].

Another compound tested to reduce mtDNA from 3a6 hMSCs was MPP^+^. This molecule inhibits mtDNA replication by preventing incorporation of 5-bromo-20-deoxyuridine into mitochondrial, but not into nuclear DNA [27,29]. Data generated by several studies suggested that MPP^+^ does not affect the overall biogenesis of mitochondria because mitochondrial transcription factor A is not decreased in treated cells. Thus, MPP^+^ selectively inhibits the replication of mtDNA, decreasing the intracellular content of mtDNA in treated cell lines [27,29,40,41]. In our work, 3a6 hMSCs could survive in the presence of MPP^+^ for only a short time. The results of the effect of MPP^+^ described in the literature demonstrated that MPP^+^ initiated lipid peroxidation, stimulated ROS production and decreased ATP levels, all processes implicated in the cell death induced by this compound [27] and indicating potent cellular toxicity in hMSCs.

The other two compounds tested (AZT and d4t) are NRTIs, a group of drugs used in the treatment of AIDS associated with lipodystrophy. The prevailing hypothesis for their toxicity suggests that these drugs inhibit mtDNA polymerase γ [26,42]. AZT inhibits thymidine phosphorylation and may deplete intracellular thymidine-5´-triphosphate (TTP) to generate an imbalance between TTP and other deoxynucleotides [43–46]. In our study with hMSCs, AZT increased mtDNA content in both 3a6 and KP cell lines, in agreement with results obtained by other authors [43,47]. Although the mechanisms through which AZT increased mtDNA are unknown, some studies have indicated that this compound up-regulates genes encoding mtDNA [48]. Other authors have suggested that the mechanism is a compensatory response to mitochondrial dysfunction, which includes inhibition of mtDNA polymerase γ [47], oxidative stress [49] and increased mitochondrial mass because of oxidative stress [50,51]. Another NRTI thymidine analogue, potentially much more toxic to polymerase γ [42,52], is Stavudine (d4t). Our results are in agreement with previous reports [43,53] that d4t induced significant reduction of the mtDNA copy number in both hMSC cell lines analyzed compared to cells without treatment. The mitochondrial copy number in 3a6 cells treated with d4t (3a6 Rho-0: 8.3±3.01) is in a similar range of values obtained with 143B.TK-Rho-0 cells (4.11±1.97); both values were calculated using the previously described methods. In addition, the low copy number of mtDNA was maintained for 288 hours after removing d4t from the hMSC culture. These data support the stability of the low number of mtDNA copies even in the absence of drug. The mitochondrial network was broken when 3a6 cells became 3a6 Rho-0 cells. The mitochondrial morphology changed and the mtDNA depletion induced mitochondrial fragmentation like that described in other Rho-0 cell lines generated by other methods [54,55]. The data obtained on mitochondrial respiration indicated that the 3a6 Rho-0 cells generated in this work have greatly impaired respiration, like that of a typical Rho-0 cell line [54,55]. All these characteristics indicate that hMSCs cultured with d4t are really Rho-0 cells.

Because the results confirm that 3a6 hMSCs can become 3a6 Rho-0 cells, it was necessary to show that these cells continue being MSCs. To confirm the MSC phenotype, cell surface receptor patterns were studied in both 3a6-wt and 3a6 Rho-0. The results showed a similar receptor pattern for four receptors in both cell types, but 3a6 Rho-0 had a lower percentage of CD105. However, this value agrees with the value for this receptor in the parental cell line (3a6) [33] and in the original cell line from which the KP cell line was obtained, which had levels of CD105 less than 20% [32]. In addition, 3a6 Rho-0 showed high levels of SSEA. Taken together, these findings suggest that hMSC Rho-0 cells still express the characteristic MSC phenotype pattern.

ROS production was assessed using flow cytometry with the results showing the same level of ROS for both 3a6-wt and 3a6 Rho-0 cells. These data differ from previous studies reporting that Rho cell lines and their parental cell lines produce different levels of ROS [56,57]. However, the ROS level of 3a6 Rho-0 cells is very similar to the ROS level of classical Rho-0 cells (143B.TK-Rho-0). It is possible that the capacity of 3a6 hMSCs to produce ROS is lower than the differentiated cell lines used in other studies [56].

Survival and the percentage of apoptosis in both 3a6-wt and 3a6 Rho-0 cell lines were similar. 3a6 Rho-0 cell growth was dependent on uridine as shown previously in other Rho-0 cell lines. The percentage of apoptotic cells with uridine exposure showed a normal number of apoptotic cells. The induction of apoptosis with staurosporine was also similar for both cell types. These data indicate that the generated cells have normal viability in culture and are suitable for study of the role of mitochondria in apoptosis.

The lower mitochondrial membrane potential (Δψm) of 3a6 Rho-0 cells compared to 3a6-wt cells agrees with the results from another Rho-0 cell line when compared with parental cells. For example, 143B.TK-Rho-0 had 36% less Δψm than 143B.TK-. However, the Δψm of other Rho-0 lines (HepG2 and HeLaS3), did not reflect differences from their parental cells [58]. Several authors have shown that Rho-0 cell lines maintain the Δψm because it is necessary for growth [59] and that the Δψm is maintained by a residual proton gradient [60–62]. Assuming that the mitochondrial electron transport chain (ETC) is the major source of intracellular ROS, the results indicated that the ETC works well in cells that had been depleted of their mtDNA, in line with the results obtained by Δψm.

The methodologies described in this work were tested in hMSCs from primary bone marrow and synovial tissue cultures. Analyses of Rd6g and d4t efficiency in hMSCs from primary cultures revealed differences in efficiency depending on the patient (data not shown). However, the most important limitation of using hMSCs from primary culture was their limited ability for expansion under standard conditions, especially with increasing age of the patient [28,29] and rapid senescence in culture [30,31].

The protocols described here allowed us to generate Rho-0 cells from hMSCs avoiding some typical inconveniences from using tumour cells, such as aneuploidy. This characteristic varies extensively among different Rho-0 cell lines [18–20] and, to date, it is not clear how aneuploidy impacts mitochondrial function. In addition, we tested several compounds and protocols to avoid using EtBr, a compound with very important toxic effects on population health. Among the compounds and conditions tested in this work, we suggest that d4t exposure at 0.5 mM for 240 hours is the best protocol to generate Rho-0 cells from 3a6 and KP hMSCs (**Tables 2** and **3**).

We also evaluated the effect of depletion of mtDNA on stem cell-like properties. Our data suggest a decrease of stem-like potentiality during mtDNA depletion. Some researchers have shown a relationship between mtDNA and the level of DNA methylation [63–65]. Recent evidence using a global assessment of DNA methylation found that cells depleted in mtDNA showed altered DNA methylation of the nuclear genome [64]. This finding may help explain our finding differences in the expression of several genes involved in MSC differentiation from 3a6-wt to 3a6 Rho-0, but additional work will be needed to confirm this.

Core pluripotency, including Nanog, Oct-4 and Sox-2, share points of convergence with STAT3, a master metabolic regulator controlling the oxidative glycolytic switch [66–69]. 3a6 Rho-0 cells have a lower expression of these genes than 3a6-wt cells. Taking into account that the transition from glycolysis to mitochondrial oxidative metabolism and maintenance of mitochondrial electron transport function are critical for differentiation [64,68], perhaps 3a6 cells will have more difficulty to respond under mesenchymal induction.

hMSCs are somatic stem cells with the capacity to differentiate into multiple lineages, including osteoblasts, adipocytes and chondrocytes. In this work we evaluated whether the mtDNA-depleted 3a6 Rho-0 cell line could differentiate into osteoblasts, adipocytes and chondrocytes. The results indicated that both 3a6-wt and 3a6 Rho-0 cell lines could be induced to differentiate under defined culture conditions, but the capacity for response was different. Osteogenic and chondrogenic differentiation was more effective in 3a6-wt than in 3a6 Rho-0 cells, while adipogenesis was more effective in 3a6 Rho-0 cells. A possible explanation could be that metabolic changes in hMSCs may differ drastically depending on which lineage they are induced to differentiate [70]. The genetic expression levels analyzed were low, probably due to the short induction time, although 9 days was described in the literature as an optimal time for evaluating the differentiation process in hMSCs. The expression of the OP gene was detected 14 days after induction, while PPAR-γ was detected 7 days after induction in the KP cell line [32]. OC expression was positive in 3a6 cells after 14 days in osteogenic medium [33]. We followed the differentiation for 9 days to analyze the differentiation capacity of 3a6 Rho-0 cells compared to 3a6 cells. Perhaps an in-depth analysis of the differentiation process under longer differentiation time would be interesting.

The different values for CD105 between 3a6-wt and 3a6 Rho-0 cells might reflect a different chondrogenic capacity of both cell types, as recently described by Fan and coworkers, who found that CD105 promotes chondrogenesis in MSCs obtained from synovium [71]. This data opens several new areas for research.

Several authors have described that during hMSC osteogenic and adipogenic differentiation there is a well coordinated process involving up-regulation of the biogenesis and respiratory function of mitochondria and expression of antioxidant enzymes. [66,67,72]. Mitochondrial biogenesis has been shown to be essential for adipogenic differentiation of the 3T3 mouse embryonic fibroblast cell line [73]. Knowing the metabolic switch during hMSC differentiation is dependent on increased mitochondrial activity, the analyses of PGC1α, a key for mitochondrial biogenesis, and TFAM, a transcription factor that binds to the mitochondrial genome, increasing their replication and mitochondrial gene expression in differentiation processes, were performed in 3a6-wt and 3a6 Rho-0 cells. PGC1α and TFAM gene expressions were increased during the differentiation process, and this increase was higher in 3a6 Rho-0 cells under adipogenic induction. This suggests that, during differentiation, an increase in mitochondrial mass could result from increased biogenesis being more significant in cells with low levels of mtDNA copy number.

This data is in line with that of other groups, who showed that when the mtDNA copy number was analyzed during differentiation, a dynamic change upon induction occurred. One explanation for this could be that down-regulation of glycolysis genes and activation of respiratory genes by PGC-1α increased the mtDNA [69,72]. The results shown here conformed to the idea that the mitochondria and bioenergetic functions play an important role in stem cells and in their differentiation process.

In summary, this is the first study to describe a protocol for the generation of Rho-0 cells using immortalized hMSCs and alternative compounds to EtBr. Because the generation of Rho-0 cells is the first step to construct cybrids, we suggest the use of hMSCs as nuclear donors. This model will allow study of the role of mitochondria as well as the different mtDNA haplogroups (or mtDNA SNPs) in the differentiation process. In addition, it will be possible to analyze the role of mitochondrial SNPs in specific cells obtained from hMSC differentiation. Several studies have shown that hMSCs are dependent on glycolysis for energy supply, but quickly shift to aerobic metabolism once they are induced to undergo differentiation. To our knowledge no one has published results like ours analyzing the mitochondrial role during the differentiation process.

## Supporting Information

S1 File **Table A**: **List of cell used in this work. Table B: Primer using in qRT-PCR.** Primers sequence using in the expression levels experiments with the corresponding Roche number probe. The gene name and the pathway that each of them is implicated are represented.
(DOCX)
