# Supplementary material for: Generating Rho-0 Cells Using Mesenchymal Stem Cell Lines
Source: PLoS One. 2016 Oct 20;11(10):e0164199. doi: 10.1371/journal.pone.0164199 (PMC5072612; doi:10.1371/journal.pone.0164199)
Supplement: S1 File — Table A: List of cell used in this work. Table B: Primer using in qRT-PCR. Primers sequence using in the expression levels experiments with the corresponding Roche number probe. The gene name and the pathway that each of them is implicated are represented. (DOCX) [file pone.0164199.s001.docx]

**Table A. List and description of cells used in the work.**

| **Cell Line** | **Origin** | **Medium** | **Source [Reference]** |
| --- | --- | --- | --- |
| **KP** | Bone marrow derived human Mesenchimal Stem Cells (hMSC) | MSC growth Medium | Hung Group [32] |
| **3a6** | Bone marrow derived human Mesenchimal Stem Cells (hMSC) | DMEM10% FBS | Hung Group [33] |
| **143B.TK-** | Osteosarcoma | DMEM10% FBS | ECACC |
| **143B.TK^-^Rho0** | Osteosarcoma | DMEM10% FBS+Uridine | Garesse Group [16] |

**Table B.** **Sequence primers and probe** **using in the amplification analysis.** Gene expression was calculated relative to the housekeeping gene (RPL13A).

| **Pathway** | **Gene** | **Fv (5´-3´)** | **Rw (5´-3´)** | **Probe (Roche)** |
| --- | --- | --- | --- | --- |
| **multipotential capacity** | Nano-HomeoBox (Nano-g) | gagatgcctcacacggagac | agggctgtcctgaataagca | #69 |
|  | POU Class 5 Homeobox 1 (Oct 3/4) | tgaagaacaagtgccaaatagc | gcggctatacaaagtggacaa | #42 |
|  | SRY (Sex determining region)-Y-box 2 (Sox-2) | gggggaatggaccttgtatag | gcaaagctcctaccgtacca | #65 |
|  | SRY-box 9 (Sox-9) | gtacccgcacttgcacaac | tcgctctcgttcagaagtctc | #61 |
| **Osteogenesis differentiation** | Alkaline phosphatase-4 (ALP) | cctgccttactaactccttagtgc | cgttggtgttgagcttctga | #37 |
|  | Osteocalcin-1 (OC-1) | ggcgctacctgtatcaatgg | tcagccaactcgtcacagtc | #33 |
| **adipogenesis differentiation** | Fatty acid synthase (FASN) | ttctgggacaacctcatcg | agacaggtccttcagcttgc | #11 |
|  | Peroxisome proliferator-activated receptor gamma (PPAR-γ) | tgagagggccaagcaaag | ataaatcacacggcgctctt | #13 |
| **Chondrogenesis differentiation** | Collagen type 1-A | ctggccccattggtaatgt | accagggaaaccagtagcac | #1 |
|  | Collagen type 2-A | cccagtctctccacgttcac | tggtgctaatggcgagaag | #4 |
| **Mitochondrial Biogenesis** | Peroxisome proliferator-activated receptor gamma co-activator 1-alpha (PGC-1α) | tgagagggccaagcaaag | ataaatcacacggcgctctt | #13 |
|  | Tanscription factor A mitochondrial (TFAM) | ctatggcctgtggatcaaatc | aaacacaattcctagctcacagg | #27 |
| **Housekeeping** | Ribosomal protein L13a | caagcggatgaacaccaac | tgtggggcagcatacctc | #28 |
